# Interassay and interlaboratory comparison of von Willebrand factor antigen as a diagnostic and prognostic biomarker in advanced chronic liver disease

**DOI:** 10.1016/j.rpth.2026.107023

**Published:** 2026-10-13

**Authors:** Lorenz Balcar, Alberto Zanetto, Jelle Adelmeijer, Benedikt Simbrunner, Elena Matthey, Cristiana Bulato, Maxime Zermatten, Kerstin Zinober, Georg Semmler, Mathias Jachs, Bernhard Scheiner, Michael Trauner, Peter Quehenberger, Thomas Reiberger, Francisco Javier Gomez, Paolo Simioni, Lorenzo Alberio, Ton Lisman, Mattias Mandorfer

**Affiliations:** 1 Division of Gastroenterology and Hepatology, Department of Medicine III, Medical University of Vienna, Vienna, Austria; 2 Vienna Hepatic Hemodynamic Lab, Division of Gastroenterology and Hepatology, Department of Medicine III, Medical University of Vienna, Vienna, Austria; 3 Clinical Research Group MOTION, Medical University of Vienna, Vienna, Austria; 4 Gastroenterology and Multivisceral Transplant Unit, Department of Surgery, Oncology, and Gastroenterology, Padova University Hospital, Padova, Italy; 5 Surgical Research Laboratory and Section of Hepatobiliary Surgery and Liver Transplantation, Department of Surgery, University of Groningen, University Medical Center Groningen, Groningen, the Netherlands; 6 Service et Laboratoire central d’hématologie, Centre Hospitalier Universitaire Vaudois (CHUV), Université de Lausanne (UNIL), Lausanne, Switzerland; 7 First Chair of Internal Medicine and Thrombotic and Haemorrhagic Disease Unit, Department of Medicine, University Hospital of Padova, Padova, Italy; 8 Department of Laboratory Medicine, Medical University of Vienna, Vienna, Austria

**Keywords:** advanced chronic liver disease, CSPH, NIT, portal hypertension, VWF

## Abstract

**Background:**

Von Willebrand factor antigen (VWF:Ag) is an inexpensive assay that is broadly available in diagnostic laboratories. The ratio of von Willebrand factor antigen and platelet count (VITRO) score (VWF:Ag/platelet count ratio) is the most promising blood-based test for clinically significant portal hypertension (CSPH) in compensated advanced chronic liver disease (cACLD). However, most published data are based on the Stago VWF:Ag assay. To allow widespread clinical application, we performed a comparative study with other commonly used assays.

**Methods:**

We included 353 patients with cACLD and decompensated cirrhosis undergoing hepatic venous pressure gradient measurement. Frozen citrated plasma samples were provided to centers with expertise in hemostasis and coagulation tests applying different assays (ie, Stago-Groningen, Werfen/IL-Padova, and Siemens-Lausanne).

**Results:**

Mean differences between VWF:Ag and VITRO levels of Stago vs Siemens were 49.6 (95% CI, 43.9-55.3) % vs 0.59 (95% CI, 0.51-0.67) and between those of Stago vs Werfen/IL were 12.1 (95% CI, 5.7-18.5) % vs 0.18 (95% CI, 0.10-0.26). Stago and Werfen/IL (intraclass correlation, 0.911; *r*, 0.914) as well as Siemens (intraclass correlation, 0.921; *r*, 0.922) showed near perfect correlation. Systematically lower VWF:Ag values by the Siemens assay are amendable by a newly developed conversion formula for patients with ACLD. Among patients with cACLD and Child-Turcotte-Pugh stage A, VITRO scores yielded consistently high areas under the curve (Stago: 0.865; Werfen/IL: 0.865; Siemens: 0.855). Using the previously established VITRO score cutoff of ≥2.5 to rule in CSPH, the positive predictive value was >90% with all assays.

**Conclusion:**

Our head-to-head comparison indicates that the widespread introduction of VWF:Ag and VITRO score into clinical practice may rely on existing infrastructure, as commonly used diagnostic assays either yielded consistent, or in case of Siemens, easily convertible results. For ruling-in CSPH in cACLD by VITRO, no conversion is required.

## Essentials

• Most published data of the von Willebrand factor antigen are based on the Stago assay.
• Interassay and interlaboratory differences in patients with cirrhosis were compared.
• Commonly used diagnostic assays yield consistent, or in case of Siemens, easily convertible results.
• No conversion for ratio of von Willebrand factor antigen and platelet count is needed to rule in clinically significant portal hypertension in compensated advanced chronic liver disease.

## Graphical abstract

**Figure:**
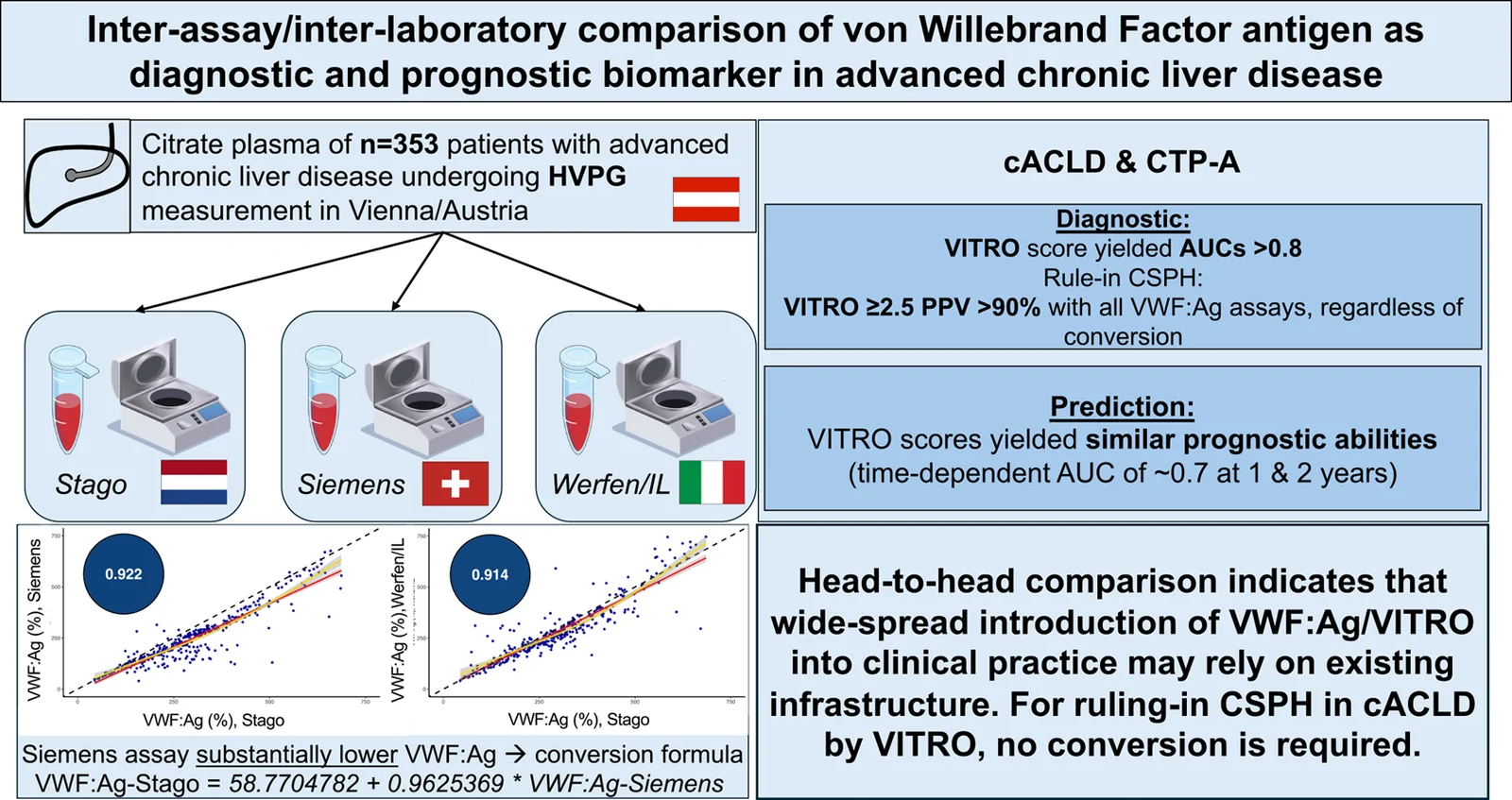

## 1 Introduction

Considering the growing evidence for the use of noninvasive tests (NITs) and the increasing availability of elastography, NITs (including blood-based tests) have been integrated into modern clinical hepatology. NITs have transformed hepatology from a field that strongly relies on histology requiring liver biopsy and other procedures such as hepatic venous pressure gradient (HVPG) measurement and endoscopy, to a modern specialty in which easily repeatable NITs guide patient management, thereby promoting precision medicine [1].

Compensated advanced chronic liver disease (cACLD) and clinically significant portal hypertension (CSPH) can be diagnosed noninvasively with important consequences in the clinical management of patients [1], [2]. However, besides elastography-based NITs for CSPH, data remain limited. The von Willebrand factor antigen (VWF:Ag), an inexpensive laboratory test, is the most promising and a potentially easily implementable blood-based test, given that it is widely available, primarily due to its role in diagnosing von Willebrand disease, the most common inherited bleeding disorder [3]. The VITRO score [4], the ratio of VWF:Ag and platelet count (PLT), may replace elastography-based NITs for ruling-in and ruling-out CSPH or reducing the diagnostic gray zone of the Baveno VII criteria [2], [5], [6]. Finally, VWF:Ag and VITRO also confer important prognostic information in patients with cACLD but also decompensated cirrhosis [7], [8], [9], [10], with the prognostic performance of VITRO being similar to that of the reference standard, HVPG [11]. VWF:Ag dynamics may also identify patients in whom nonselective β-blocker treatment is particularly beneficial [12].

However, the vast majority of published results linking the VITRO score or VWF:Ag levels to CSPH and clinical outcome are based on the Stago VWF:Ag assay, that is, only one of the major manufacturers used in clinical routine laboratories. VWF:Ag assays have been developed for diagnosing specific forms of von Willebrand disease, in which usually low levels of VWF:Ag are detected. Contrarily, VWF:Ag is commonly elevated in patients with ACLD, and individual VWF:Ag assays have not been systematically compared in this context. Finally, values often exceed the upper limit of quantification of the respective assays, and additional dilutions may be used to estimate high values. Although the latter may improve the prognostic value of VWF:Ag, dilutions are inconsistently applied [13]. Therefore, a comparative study of VWF:Ag across different assays and laboratories is required before promoting its widespread clinical application in ACLD/cirrhosis [14]. Similar considerations apply to VITRO, which is calculated from VWF:Ag.

The objective of this study was therefore to conduct a comparative analysis of Stago with the other commonly used assays (ie, Werfen/IL and Siemens) in order to evaluate interassay and interlaboratory differences and VWF:Ag assay laboratory-specific diagnostic and prognostic performances. Moreover, implications for the diagnostic and prognostic performances of VITRO are analyzed.

## 2 Methods

### 2.1 Study design and patients

We analyzed citrate plasma samples from 353 patients with ACLD undergoing HVPG measurement at the Vienna Hepatic Hemodynamic Lab within the prospective Vienna cirrhosis study (VICIS, NCT03267615). The inclusion criterion was HVPG ≥6 mm Hg. Patients were excluded if any of the following criteria were present: patients with a history of liver transplantation, vascular liver diseases, any active extrahepatic malignancies, hepatocellular carcinoma outside of Milan criteria, missing information on important laboratory parameters and/or clinical follow-up, and insufficient amount of citrate plasma available. Based on previous findings, patients on statins were excluded [15]. The prospective cohort included patients enrolled between December 2016 and November 2023 (Supplementary Figure 1). Study-relevant clinical and laboratory information was collected from patients’ medical records.

### 2.2 Measurement of biomarkers

Blood samples were obtained via a central venous line (ie, the side port of the catheter introducer sheath) at the time of HVPG measurement. Stored frozen samples were provided to centers with expertise in hemostasis and coagulation tests and academic interest in coagulation in liver disease applying different VWF:Ag assays (ie, Groningen: Stago, Padova: Werfen/IL and Stago, and Lausanne: Siemens). Groningen used the Stago assay on an STA Compact Max 3 analyzer (Stago) and Lausanne used the Siemens assay (VWF:Ag assay; Siemens) and analyzer (CN6000; Sysmex), whereas Padova performed the VWF:Ag measurements by the Werfen/IL (HemosIL AcuStar VWF:Ag; Werfen/Instrumentation Laboratory) and analyzer (ACL AcuStar analyzer; Instrumentation Laboratory).

Regarding sample dilution, the Stago assay (Groningen) is linear until 100% and automatically dilutes samples with higher values to receive a linear calibration curve. Samples were manually prediluted to save reagents. According to the standard application protocol, the VWF-Ag assay (Siemens) performed on a CN6000 analyzer (Sysmex) does not permit to extrapolate results above the highest caliration point. Therefore, plasma with values above the highest calibration point (232% with the calibration curve used during the study) was reanalyzed after a 1:8 dilution (in Owren buffer). For VWF:Ag determination using the HemosIL AcuStar (Werfen), the assay linearity range is 0.3% to 400%. For values above 400%, the instrument automatically performs a 1:20 dilution of the sample. No manual sample dilutions were performed.

The respective VITRO scores (ie, the VWF:Ag/PLT ratio [4]) were calculated for each assay individually.

### 2.3 HVPG measurement

HVPG measurement was performed according to a local standardized operating procedure [16], which is in line with national [2], [5] and international consensuses [1], [17]. Under local anesthesia and using ultrasound guidance, a catheter introducer sheath was inserted into the right internal jugular vein. Subsequently, a hepatic vein was cannulated by a specifically designed balloon catheter, and the free and wedged hepatic venous pressures were obtained and recorded at least as triplicate measurements. CSPH was defined by a threshold of HVPG ≥10 mm Hg.

### 2.4 Statistical analysis

All statistical analyses were performed using R (version 4.4.2; R Core Team, R Foundation for Statistical Computing). Categorical variables were reported as absolute (*n*) and relative frequencies (%), whereas continuous variables as mean ± SD or median (IQR), as appropriate. Student’s *t*-test was used for group comparisons of normally distributed variables and the Mann-Whitney U-test for non-normally distributed variables. Group comparisons of categorical variables were performed using either chi-squared or Fisher’s exact test, as appropriate.

Frozen samples were compared. VWF:Ag levels from different assays were graphically compared using dot and density plots with VWF:Ag levels from the Stago assay (ie, Stago assay on a Stago analyzer) as a reference. Linear regression and intraclass correlation coefficient (ICC) analyses were used to evaluate the intra-assay relationship and their correlation. Calibration was assessed as the mean difference (ie, Bland-Altman analysis) to assess internal agreement, including the mean difference (ie, bias ≙ calibration in the large) and the lower and upper limits of agreement (ie, 95% CI of agreement corresponding to ±1.96 × SD of observed difference). Also, histograms of residuals and Q-Q plots were generated. Calibration slope and intercept were derived from the linear regression model. Moreover, calibration was investigated graphically by using natural splines with 3 degrees of freedom and inspecting the deviation from the linear regression line. *R*^2^ (ie, variation and proportion of variation) and Pearson’s correlation coefficient *r* were provided.

As Siemens values yielded systematically lower absolute levels of VWF:Ag, a conversion formula was derived. Due to the nonlinear relationship between the Siemens and the Stago-Groningen VWF:Ag levels, a polynomial model was used next to a linear model to create a conversion formula.

Paired comparisons were analyzed using McNemar's test with and without Holm correction. In addition, agreement between assays was quantified using Cohen's kappa and the overall proportion of agreement.

The area under the receiver operating characteristics curve (AUROC) of different VWF:Ag assays for CSPH was calculated using the pROC package in patients with cACLD and Child-Turcotte-Pugh (CTP) stage A as well as for to the VITRO score [4] and Baveno VII criteria [1].

Hepatic decompensation was defined as the first occurrence of clinically apparent ascites, variceal bleeding, or overt hepatic encephalopathy [1]. The median follow-up was calculated using the reverse Kaplan-Meier method. Time-dependent AUROCs for the prediction of hepatic decompensation during the first 2 years of follow-up were computed using the timeROC package. Similarly, analyses were calculated for patients with decompensated cirrhosis. Time-dependent AUROCs were calculated and Cox regression analyses were performed, with further decompensation and/or liver-related death as outcomes of interest.

The level of significance was set at a 2-sided *P* value of <.05.

### 2.5 Ethics

This study has been conducted in accordance with the principles of the Declaration of Helsinki and its amendments and has been approved by the local ethics committee, which waived the requirement of written informed consent.

## 3 Results

### 3.1 Patient characteristics

After applying inclusion and exclusion criteria, 353 patients were included (Figure 1, Table 1). The detailed patient characteristics can be found in Supplementary Table 1.

**Figure 1:**
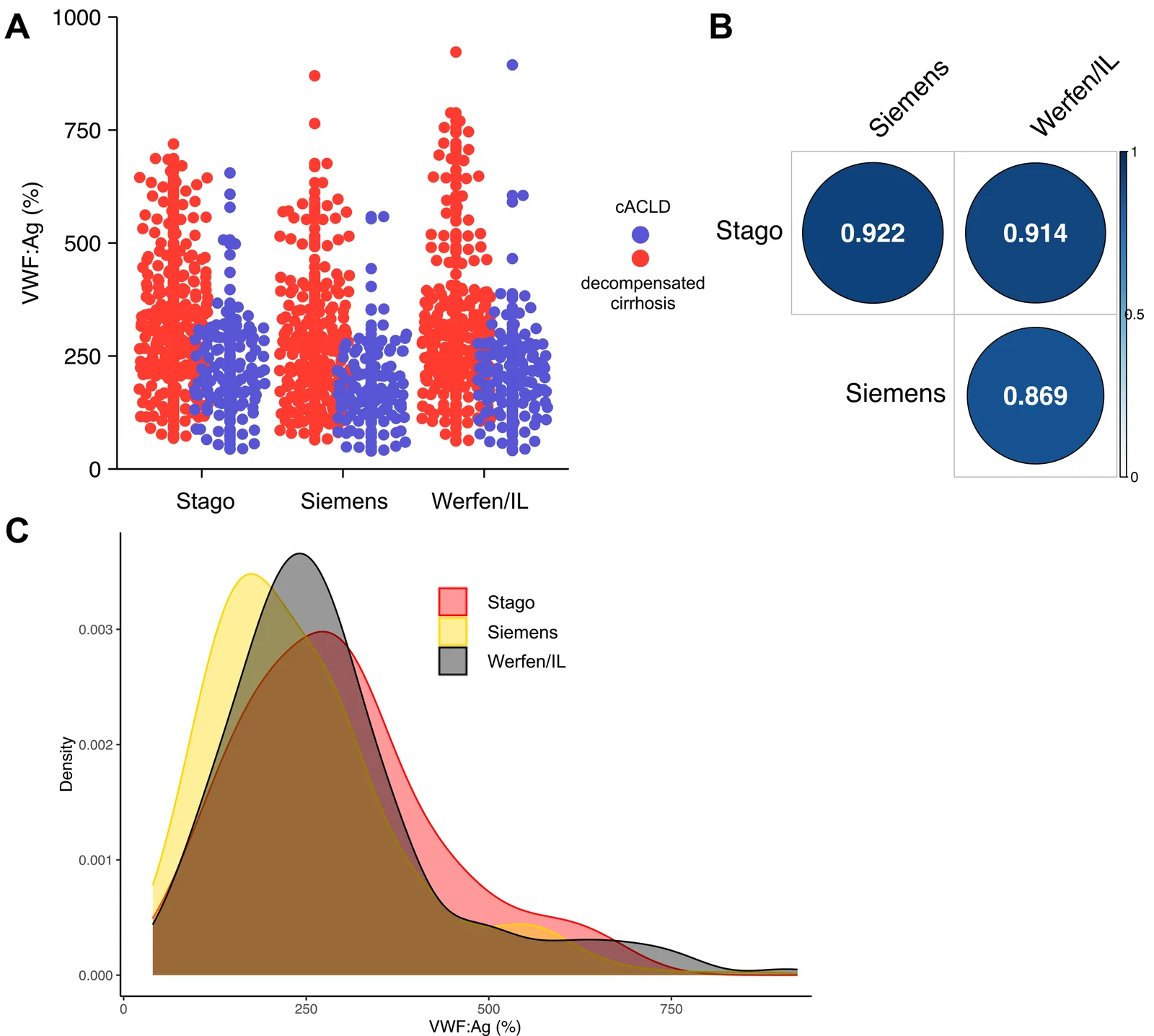
(A) Dot plots comparing VWF:Ag levels according to different assays. (B) Intraclass correlation coefficients of VWF:Ag levels measured with different assays. (C) Density plots comparing the distribution of VWF:Ag levels according to different assays. cACLD, compensated advanced chronic liver disease; VWF:Ag, von Willebrand factor antigen.

**Table 1:** Patient characteristics.

Patient characteristics	Overall (*n* = 353)
Age (y), mean (SD)	56.2 (11.9)
Sex, *n* (%)	
Male	243 (68.8)
Female	110 (31.2)
Etiology of liver disease, *n* (%)	
Alcohol-related liver disease	175 (49.6)
Viral	61 (17.3)
Alcohol-related liver disease/viral	25 (7.1)
Metabolic dysfunction–associated steatotic liver disease	19 (5.4)
Cholestatic	18 (5.1)
Other etiologies	55 (15.6)
Anticoagulation at study inclusion, *n* (%)	14 (4.0)
Antiplatelet therapy at study inclusion, *n* (%)	24 (6.8)
Hepatocellular carcinoma within Milan criteria, *n* (%)	32 (9.1)
Characterization of portal hypertension	
History of decompensation, *n* (%)	232 (65.7)
Hepatic venous pressure gradient (mm Hg), median (IQR)	17.0 (12.0-21.0)
Clinically significant portal hypertension, *n* (%)	310 (87.8)
Liver disease scores	
Child-Turcotte-Pugh score (points), median (IQR)	7 (5-9)
A, *n* (%)	149 (42.2)
B, *n* (%)	147 (41.6)
C, *n* (%)	57 (16.1)
MELD score (points), median (IQR)	12 (9-16)
MELD-Na score (points), median (IQR)	14 (10-18)
UNOS MELD (2016) score (points), median (IQR)	12 (9-16)
VWF:Ag levels and VITRO scores, median (IQR)	
VWF:Ag, Stago (%)	282 (195-358)
VWF:Ag, Siemens (%)	215 (148-298)
VWF:Ag, Werfen/IL (%)	256 (193-325)
VITRO, Stago	2.75 (1.61-4.47)
VITRO, Siemens	2.20 (1.31-3.72)
VITRO Werfen/IL	2.48 (1.54-4.19)

MELD score, model for end-stage liver disease; *n*, number; UNOS, United Network for Organ Sharing; VITRO, ratio of von Willebrand factor antigen and platelet count; VWF:Ag, von Willebrand factor antigen.

The median VWF:Ag with the Stago assay was 282 (IQR, 195-358) % compared with 215 (IQR, 148-298) % with the Siemens assay and 256 (IQR, 193-325) % with the Werfen/IL assay (Table 1, Figure 1A). This translated into VITRO values of 2.75 (IQR, 1.61-4.47) using the Stago assay, 2.20 (IQR, 1.31-3.72) for the Siemens assay, and 2.48 (IQR, 1.54-4.19) for the Werfen/IL assay (Supplementary Table 1). Median VWF:Ag and VITRO levels according to disease stage are provided in Supplementary Table 2.

### 3.2 Correlation, calibration, and agreement between different VWF:Ag assays, using Stago (Groningen) as the reference test

VWF:Ag Stago vs Siemens (Pearson’s *r* = 0.922; ICC, 0.921) and Stago vs Werfen/IL (Pearson’s *r* = 0.914; ICC, 0.911) showed excellent correlations (Figure 1B). However, the Siemens assay found consistently lower VWF:Ag values, when considering the Stago assay as the reference test (Figure 1C).

The relationships between Stago and the other studied VWF:Ag were not strictly linear (Figure 2). Agreement between Stago and the other studied VWF:Ag assays was assessed using the Bland-Altman method and plot (Figure 3). The mean difference between assays indicated systematically lower values, in particular for the Siemens assay (49.6 [95% CI, 43.9-55.3] %; Werfen/IL: 12.1 [95% CI, 5.7-18.5] %), and tended to be larger at higher values (Table 2, Supplementary Figure 2, Supplementary Table 3). Due to the significant difference in individual VWF:Ag levels between the Siemens and Stago assays and potential miscalibration, a conversion formula from Siemens × Stago was calculated. VWF:Ag from Siemens and Stago showed a nonlinear relationship (*P* < .001), which is why a polynomial model was created (Supplementary Figure 3). However, due to the small difference in absolute values, an additional linear model including a conversion formula was derived: VWF:Ag-Stago = (0.96 × VWF:Ag-Siemens) + 58.77. Respective Bland-Altman plots and scatter plots with nonlinear and linear fits can be found in Supplementary Figure 3.

**Figure 2:**
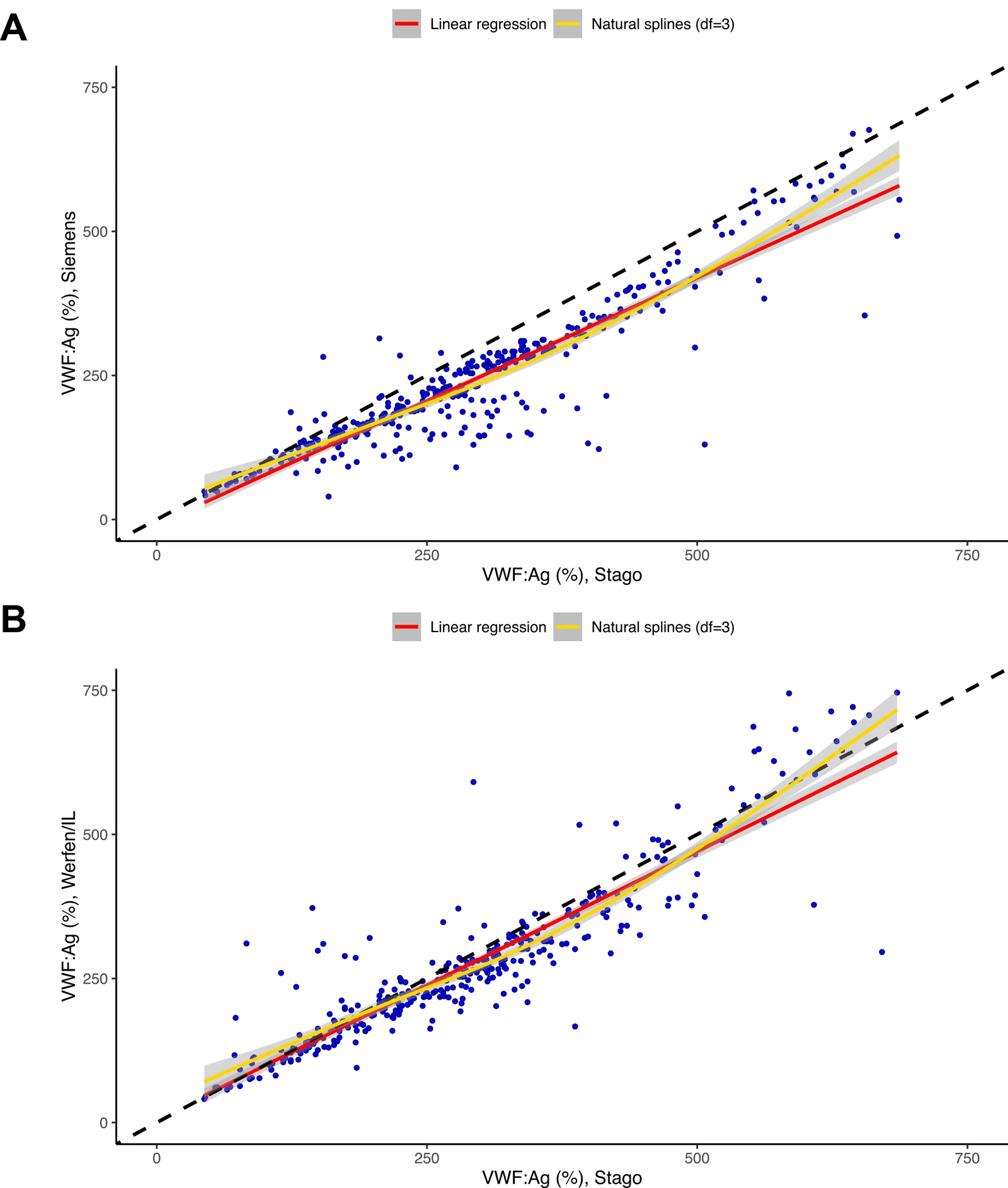
Scatterplot between the VWF:Ag values by the (A) Siemens (Lausanne) and the (B) Werfen/IL (Padova) assay on the *y*-axis and the Stago (Groningen) assay on the *x*-axis (reference). df, degrees of freedom; VWF:Ag, von Willebrand factor antigen.

**Figure 3:**
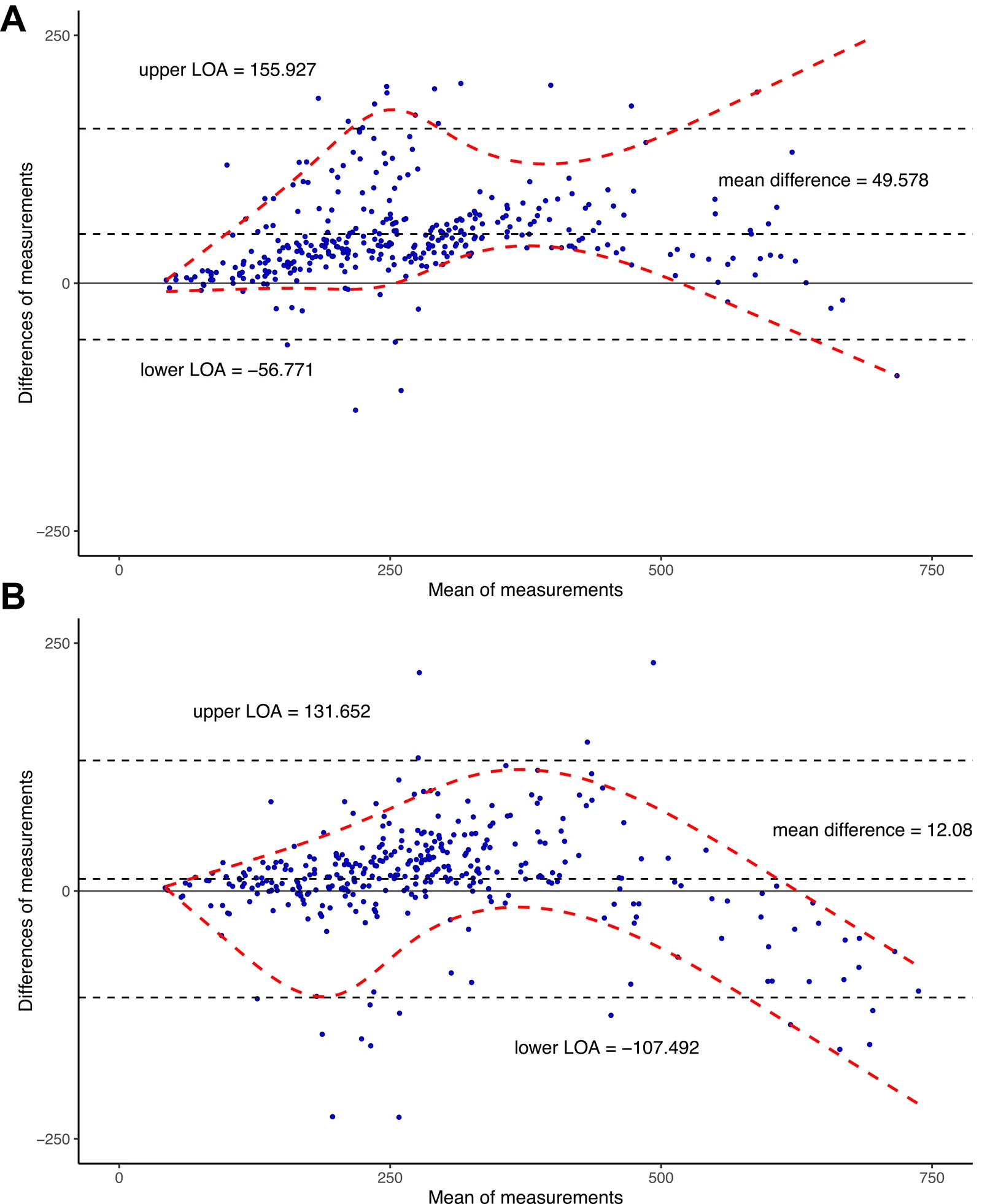
Modified Bland-Altman plots showing the mean of von Willebrand factor antigen values within a patient (*x*-axis) and the difference (Stago/reference—Siemens/Werfen/IL, *y*-axis) comparing the (A) Siemens and the (B) Werfen/IL assay with the Stago assay (reference). The dashed black lines represent the mean difference of both measurements (ie, calibration-in-the-large, "bias") and the upper and lower limits of agreement, corresponding to the area of agreement within ±1.96 times the SD of the difference observed in the respective sample. Values close to 0 indicate no difference within the same subject (ie, agreement). The red dashed lines represent quantile regression of the 95% CI of differences – means, modeled with natural splines (df = 3). df, degrees of freedom; LOA, limit of agreement.

**Table 2:** Inter-assay comparison of VWF:Ag assessing agreement between Stago (reference) and Siemens or Werfen/IL assays.

Metric	Siemens	Werfen/IL
ICC (95% CI)	0.921 (0.903-0.935)	0.911 (0.892-0.927)
Pearson’s *r*	0.922	0.914
Mean difference (95% CI) ≙ mean calibration	49.6 (43.9, 55.3)	12.1 (5.7, 18.5)
Interval of agreement	212.7	239.1
Lower limit of agreement (95% CI)	–56.8 (–66.5, –47.1)	–107.5 (–118.4, –96.6)
Upper limit of agreement (95% CI)	155.9 (146.2, 165.6)	131.7 (120.7, 142.6)
Residuals, median	–9.65	0.17

Intraclass correlation coefficient quantifies the degree of agreement between measurements. Pearson’s *r* measures the strength and direction of a linear relationship between continuous variables. The mean difference, interval of agreement, lower and upper limit of agreement, and residuals are measures derived from the Bland-Altman plot, assessing the agreement between 2 different measurement methods by measuring the difference between the 2 measurements against their average.

ICC, intraclass correlation coefficient; VWF:Ag, von Willebrand factor antigen.

### 3.3 Diagnosis of CSPH in cACLD by different VWF:Ag assays

Among 97 compensated patients with CTP stage A, the CSPH prevalence was 71.7% (Table 3). Although all assays yielded comparable AUROCs, the Stago VWF:Ag assay had the highest discriminative ability for CSPH (0.753; Werfen/IL: 0.718 Siemens: 0.712; Figure 4A). Areas under the curve (AUCs) of all VITRO versions were >0.8 (VITRO-Stago: 0.865, VITRO-Werfen/IL: 0.865, and VITRO-Siemens: 0.855; Figure 4B). Using the previously established VITRO score cutoff of ≥2.5 to rule in CSPH, the positive predictive value (PPV) was >90% with all assays; however, the Siemens assay ruled in CSPH in a slightly lower proportion of patients (Table 3). The conversion formulas (linear and nonlinear) may improve the diagnosis of CSPH using the Siemens assay by increasing sensitivity and the negative predictive value, without compromising specificity/the PPV. Importantly, the previously established VITRO thresholds at ≤1.0 to rule out and ≥2.5 to rule in CSPH showed a numerically lower gray zone, as compared with the Baveno VII criteria regardless of assay or conversion, confirming its robust performance.

**Table 3:** Predictive ability of VWF:Ag/VITRO levels for clinically significant portal hypertension (CSPH) according to the used assay in patients with cACLD and Child-Turcotte-Pugh stage A.

CSPH	Stago (ref)	Siemens	Werfen/IL
AUROC (95% CI)	0.753 (0.644-0.861)	0.712 (0.597-0.826)^a^	0.718 (0.607-0.830)^a^
	VITRO w/Stago (ref)	VITRO w/Siemens	VITRO w/Werfen/IL
AUROC (95% CI)	0.865 (0.788-0.943)	0.855 (0.773-0.937)^a^	0.865 (0.790-0.940)^a^

Decision rules	Ruled-out, *n* (%)	Sens (%)	NPV (%)	Gray zone, *n* (%)	Ruled-in, *n* (%)	Spec (%)	PPV (%)
Stago, VITRO ≤1.0/≥2.5	20 (21)	95.7	85.0	37 (38)	40 (41)	89.3	92.5
Siemens, VITRO ≤1.0/≥2.5	27 (28)	87.0	66.7	41 (42)	29 (30)	96.4	96.6
Siemens after linear conversion, VITRO ≤1.0/≥2.5	13 (13)	97.1	84.6	44 (45)	40 (41)	89.3	92.5
Siemens after nonlinear conversion, VITRO ≤1.0/≥2.5	16 (16)	95.7	81.3	42 (43)	39 (40)	92.9	94.9
Werfen/IL, VITRO ≤1.0/≥2.5	21 (22)	91.3	71.4	39 (40)	37 (38)	92.9	94.6
Baveno VII (LSM <15 kPa and PLT >150 G/L/LSM ≥25 kPa)	12 (12)	95.7	75.0	49 (51)	36 (37)	89.3	91.7

Comparison	Agreement (%)	Cohen’s kappa	Paired McNemar	Holm-adjusted *P*
VITRO ≥2.5 and CSPH, Stago vs Siemens	90.7	0.794	**0.008**	**.02**
VITRO ≥2.5 and CSPH, Siemens vs Werfen/IL	92.8	0.836	0.683	.68
VITRO ≥2.5 and CSPH, Stago vs Werfen/IL	93.8	0.868	**0.023**	**.047**

AUROC for the prediction of CSPH using the Stago assay as reference. Estimation of difference according to the De-Long test. Model characteristics for rule-in/rule-out of CSPH according to distinct cutoffs. Paired binary classifications were compared using McNemar's test with and without Holm correction. In addition, agreement between assays was quantified using Cohen's kappa and the overall proportion of agreement.

Bold indicates *P*-values <.05.

AUROC, area under the receiver operating curve; cACLD, compensated advanced chronic liver disease; LSM, liver stiffness measurement; NPV, negative predictive value; PLT, platelet count; PPV, positive predictive value; VITRO, ratio of von Willebrand factor antigen and platelet count; VWF:Ag, von Willebrand factor antigen.

a De-Long test >.05.

**Figure 4:**
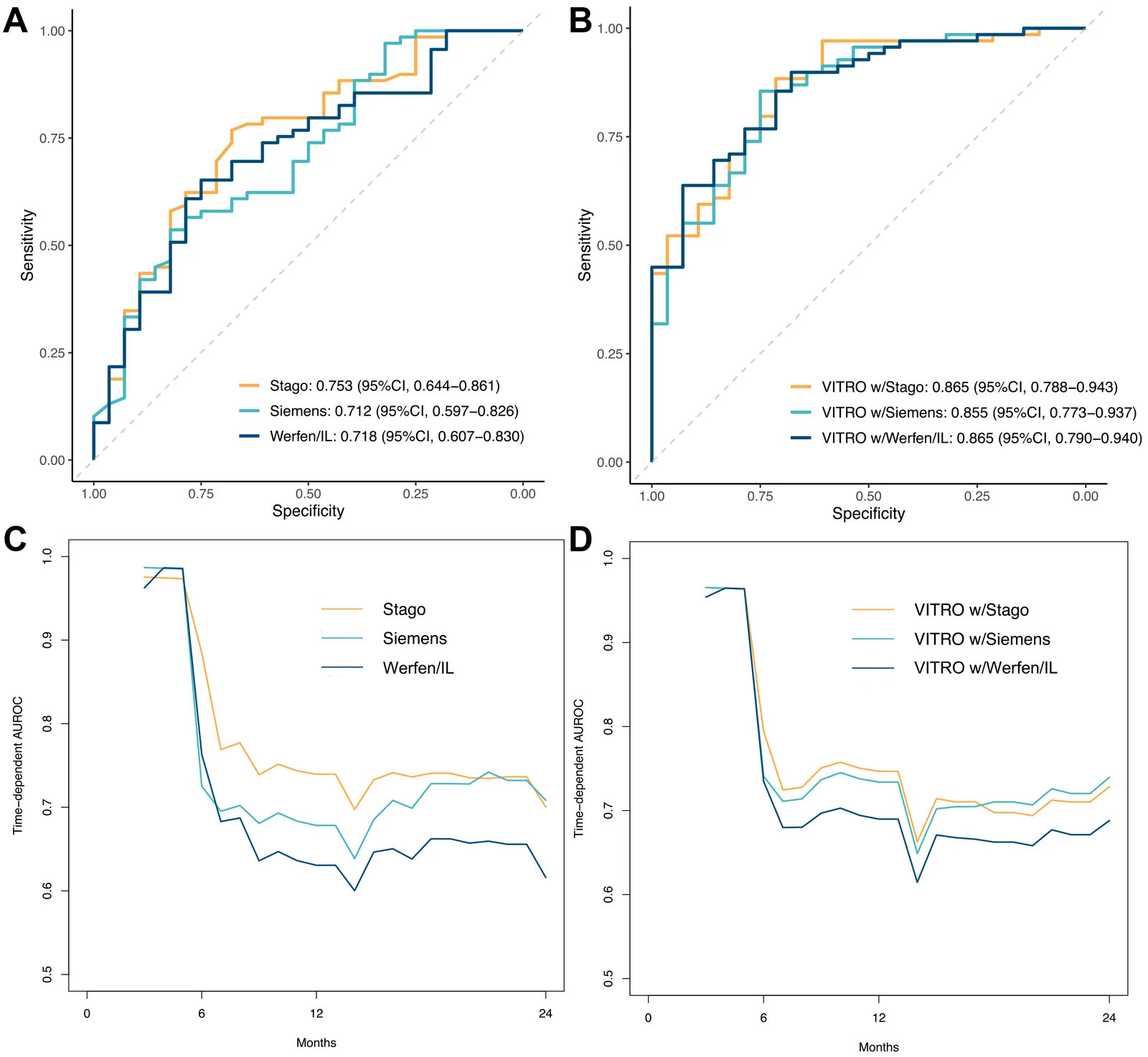
Areas under the curve of the (A) von Willebrand factor antigen values and (B) VITRO scores according to different assays to discriminate between patients with cACLD with Child-Turcotte-Pugh stage A with or without clinically significant portal hypertension as well as respective time-dependent AUROCs (C and D) to predict first hepatic decompensation. AUROC, area under the receiver operating curve; VITRO, ratio of von Willebrand factor antigen and platelets.

When applying the different assays for the VITRO cutoff to rule in CSPH in patients with cACLD and CTP-A, there was a moderate agreement between assays (Cohen's kappa ranging from 0.79 to 0.87). Pairwise McNemar tests revealed significant differences in the levels of pairs of patients classified as CSPH between Stago and Siemens (*P* = .008, after Holm correction *P* = .02) as well as between Stago and Werfen/IL (*P* = .02, after Holm correction *P* = .047). Nevertheless, agreement was high overall, ranging from 90.7% to 93.8%.

### 3.3 Prediction of hepatic decompensation in cACLD by different VWF:Ag assays

In 97 compensated patients with CTP stage A, 7 and 12 patients developed first hepatic decompensation after 12 and 24 months, respectively. Despite the low number of events during follow-up, VWF:Ag values derived from the Stago and the Siemens assays, as well as the respective VITRO scores, were significantly associated with first hepatic decompensation (Table 4). The discriminative ability of VWF:Ag determined by the Werfen/IL assay was lower (VWF:Ag Werfen/IL vs Stago: AUC 0.639 vs 0.727, *P* = .02; time-dependent AUROC at 24 months for VWF:Ag Werfen/IL vs Stago: AUROC 0.616 vs 0.700 Stago, *P* = .04; Table 4; Figure 4A, C). A specific conversion formula for this did not improve prognostication (Supplementary Figure 4). Importantly, these differences disappeared when calculating the VITRO score (Table 4; Figure 4B, D), which showed a consistent prognostic performance, regardless of the VWF:Ag assay.

**Table 4:** Predictive ability of VWF:Ag/VITRO levels for first hepatic decompensation in patients with cACLD and Child-Turcotte-Pugh stage A (*n* = 97) according to the applied assay.

First hepatic decompensation	Stago (ref)	Siemens	Werfen/IL
HR (95% CI), per 10	1.07 (1.02-1.12), *P* = .01	1.07 (1.02-1.12), *P* = .01	1.04 (0.99-1.10), *P* = .01
Concordance (SE)	0.727 (0.059)	0.718 (0.062)^a^	0.639 (0.071)^b^
Time-dependent AUROC		12 months	24 months
Events		7	12
Stago (ref)		0.740	0.700
Siemens		0.678^a^	0.709^a^
Werfen/IL		0.631^a^	0.616^c^

First hepatic decompensation	VITRO-Stago (ref)	VITRO-Siemens	VITRO-Werfen/IL
HR (95% CI), per 10	1.27 (1.04-1.54), *P* = .02	1.30 (1.05-1.61), *P* = .02	1.23 (0.99-1.53), *P* = .06
Concordance (SE)	0.703 (0.063)	0.709 (0.065)^a^	0.663 (0.067)^a^
Time-dependent AUROC		12 months	24 months
Events		7	12
VITRO-Stago (ref)		0.747	0.728
VITRO-Siemens		0.734^a^	0.740^a^
VITRO-Werfen/IL		0.690^a^	0.688^a^

AUROC, area under the receiver operating curve; cACLD, compensated advanced chronic liver disease; VITRO, ratio of von Willebrand factor antigen and platelet count; VWF:Ag, von Willebrand factor antigen.

a De-Long test *P* value >.05.

b De-Long test *P* value .02.

c De-Long test *P* value .04.

### 3.4 Prediction of further hepatic decompensation and/or liver-related death in decompensated cirrhosis by different VWF:Ag assays

In 232 decompensated patients, 75/94 patients developed further decompensation and/or liver-related death after 12/24 months, respectively. In brief, there was no difference between the assays (Supplementary Table 4). Detailed information is displayed in the Supplementary Materials (Supplementary Table 4).

## 4 Discussion

Although VWF:Ag testing is broadly available in clinical practice, among patients with ACLD/cirrhosis, VWF:Ag levels seem to vary substantially according to the type of assay.

Based on the findings of an external validation cohort from a recent study from our group (ie, not a head-to-head comparison) [7], levels of patients with cACLD recruited in Padova (Werfen/IL assay and analyzer) seemed to be significantly lower than ours (Stago assay and analyzer; 237 [IQR, 187-299] vs 300 [IQR, 222-392] %), with implications for the prognostic performance of VWF:Ag (outcome: first hepatic decompensation and/or liver-related death, Vienna: Brier score 0.092  ±  0.014 vs Padova: 0.144  ±  0.034). Accordingly, other studies using commonly applied assays (Siemens [18] and Werfen/IL [19]) on automated analyzers also seemed to yield significantly lower levels compared with those obtained by the Stago assay, although differences between individual studies and populations seem hard to interpret as VWF:Ag values depend—among other factors—on liver disease severity, which may vary. This underlines the importance of a head-to-head comparison, as performed in our interassay and interlaboratory study before relying on VWF:Ag and its derivative, VITRO, for clinical decision-making. Notably, VITRO has recently been included in a national consensus [2] as well as a European Association for the Study of the Liver Clinical Practice Guideline [20], suggesting increasing clinical use and underlining the timeliness of our study.

Therefore, in the present study, we evaluated the interassay and interlaboratory differences in VWF:Ag testing and made the following observations.

First, even in head-to-head comparison, VWF:Ag values in patients with ACLD/cirrhosis vary when using different assays. Although this may compromise model calibration and affect the diagnostic/prognostic utility of thresholds that have been proposed for clinical decision-making, the calculation of VITRO mitigated this potential limitation. VITRO calculated from different VWF:Ag assays showed a similar discriminative ability for CSPH and first hepatic decompensation in patients with cACLD with CTP stage A, that is, the key target population of NITs for CSPH. Since the Siemens assay led to systematically lower values, a conversion formula (ie, from VWF:Ag-Siemens to VWF:Ag-Stago) was developed. Notably, the diagnostic performance of VITRO calculated using VWF:Ag derived from the VWF:Ag-Siemens to VWF:Ag-Stago conversion formula (ie, [0.96 × VWF:Ag-Siemens] + 58.77) indicated a slightly increased sensitivity for CSPH in cACLD and CTP stage A, whereas specificity and PPV remained adequate. Moreover, the proportion of unclassifiable patients was numerically decreased when applying the conversion formula. Nevertheless, these changes were minor and likely clinically insignificant. Importantly, VITRO showed a robust discriminative ability of >0.8 for CSPH, regardless of the used assay, and VITRO-based criteria (≥2.5 to rule in and ≤1 to rule out CSPH) yielded a numerically smaller diagnostic gray zone as compared with the Baveno VII criteria. Thus, our interassay/interlaboratory analysis confirms that the blood-based VITRO score is a veritable alternative to elastography.

Notably, VWF:Ag values from the Werfen/IL assay provided lower prognostic utility compared with the Stago assay (AUC, 0.639 vs 0.727; *P* = .02), which is in line with the findings of the above-mentioned study [7]; however, the difference diminished after calculating the VITRO score.

Although our study has important strengths, some limitations have to be acknowledged. Patients were prospectively included at the time of HVPG measurement. We cannot rule out selection bias; however, hemodynamic evaluation of our patients is performed routinely for risk stratification and treatment monitoring purposes, and thus, we are convinced that our study population is not highly selected. All samples were obtained via a central venous line at the time of HVPG measurement, and hence, data are derived from central venous blood and not peripherally measured blood. Based on previous findings, patients on statins were excluded [15], limiting the clinical applicability of VWF:Ag and VITRO in the context of cACLD, in particular, in the light of the increasing prevalence of metabolic dysfunction and cardiovascular risk factors and MASLD and MetALD. Elastography-based models such as ANTICIPATE ± NASH and NICER show an excellent performance in the latter context [21], [22] and should be preferably used. For VITRO scores, interlaboratory differences regarding PLT could not be evaluated for logistical reasons. We want to emphasize that our study may only be applying for subjects of European descent, as they represent the vast majority of included patients. Finally, our study does not provide a methodological explanation for the systematic interassay differences. Differences in dilution strategies are not the cause of the differences provided in the results. All tests use linear calibration curves with automated dilution when a sample is outside of the curve. In diagnosing von Willebrand disease, these assays are usually congruent, so that the discrepancy between the Siemens and other assays seems to be limited to high VWF:Ag values. However, also in the diagnosis of von Willebrand disease, differences might be possible due to the use of different calibrators or antibodies. VWF is a multimeric protein and by the quantitative measurement of VWF:Ag, multimers of various sizes in plasma are measured, which is inherently difficult and could explain subtle but systematic interassay differences.

In conclusion, our head-to-head comparison indicates that the wide-spread introduction of VWF:Ag and VITRO into clinical practice may rely on existing infrastructure, as commonly used diagnostic assays either yielded consistent, or in case of Siemens, easily convertible results. For ruling-in CSPH in patients with cACLD by VITRO, no conversion is required.
