# Supplementary material for: Interassay and interlaboratory comparison of von Willebrand factor antigen as a diagnostic and prognostic biomarker in advanced chronic liver disease
Source: Res Pract Thromb Haemost. 2026 Oct 13;10(7):107023. doi: 10.1016/j.rpth.2026.107023 (PMC13760105; doi:10.1016/j.rpth.2026.107023)
Supplement: Supplementary file 1 [file mmc1.docx]

**Inter-assay and inter-laboratory comparison of von Willebrand Factor antigen as diagnostic and prognostic biomarker in advanced chronic liver disease**

Lorenz Balcar ^1,2,3^, Alberto Zanetto ^4^, Jelle Adelmeijer ^5^, Benedikt Simbrunner ^1,2,3^, Elena Matthey ^6^, Cristiana Bulato ^7^, Maxime Zermatten ^6^, Kerstin Zinober ^1^, Georg Semmler ^1,2,3^, Mathias Jachs ^1,2,3^, Bernhard Scheiner ^1,2,3^, Michael Trauner ^1,2,3^, Peter Quehenberger ^8^, Thomas Reiberger ^1,2,3^, Francisco Javier Gomez ^6^, **Paolo Simioni ^7^, Lorenzo Alberio ^6^, Ton Lisman ^5^, Mattias Mandorfer ^1,2,3^**

Authors in bold share last and corresponding authorship.

^1^ Division of Gastroenterology and Hepatology, Department of Medicine III, Medical University of Vienna, Vienna, Austria; ^2^ Vienna Hepatic Hemodynamic Lab, Division of Gastroenterology and Hepatology, Department of Medicine III, Medical University of Vienna, Vienna, Austria; ^3^ Clinical Research Group MOTION, Medical University of Vienna, Vienna, Austria; ^4^ Gastroenterology and Multivisceral Transplant Unit, Department of Surgery, Oncology, and Gastroenterology, Padova University Hospital, Padova, Italy; ^5^ Surgical Research Laboratory and Section of Hepatobiliary Surgery and Liver Transplantation, Department of Surgery, University of Groningen, University Medical Center Groningen, Groningen, The Netherlands; ^6^ Service et Laboratoire central d'hématologie, Centre Hospitalier Universitaire Vaudois (CHUV), Université de Lausanne (UNIL), Switzerland; ^7^ First Chair of Internal Medicine and Thrombotic and Haemorrhagic Disease Unit, Department of Medicine, University Hospital of Padova, Padova, Italy; ^8^ Department of Laboratory Medicine, Medical University of Vienna, Vienna, Austria

**SUPPLEMENTARY RESULTS**

**Patient characteristics**

The mean age was 56±12 years, with a male predominance (n=243, 68.8%) and alcohol-related liver disease (n=175, 49.6%) as most common aetiology of ACLD. Two-thirds of included patients (n=232, 65.7%) were decompensated and the median HVPG was 17 (IQR: 12-21) mmHg. The median CTP score was 7 (IQR: 5-9) points (CTP-A n=149, 42.2%; CTP-B n=147, 41.6%; CTP-C n=57, 16.1%). Detailed patient characteristics are displayed in Supplementary Table 1.

**SUPPLEMENTARY FIGURES**

**Supplementary Figure 1**

**Supplementary Figure 1.** Study flowchart illustrating patient selection.

*Abbreviations: ACLD, advanced chronic liver disease; HCC, hepatocellular carcinoma; HVPG, hepatic venous pressure gradient; PSVD, porto-sinusoidal vascular disorder; TIPS, transjugular intrahepatic portosystemic shunt*

**Supplementary Figure 2**

**Supplementary Figure 2.** Graphical depiction comparing the residuals of the Bland-Altman plot of the **A**) Siemens and the (**B**) Werfen/IL assay with the Stago assay (reference) as well as Q-Q plots.

*Abbreviations: VWF:Ag, von Willebrand factor antigen*

**Supplementary Figure 3**

**Supplementary Figure 3.** (**A**) Scatterplot with linear vs non-linear spline. Conversion formulas for Siemens x Stago and respective Bland-Altman plots using the (**B**) linear and (**C**) non-linear/polynomial model.

*Abbreviations: VWF:Ag, von Willebrand factor antigen*

**Supplementary Figure 4**

**Supplementary Figure 4.** (**A**) Scatterplot with linear vs non-linear spline. Conversion formulas for Werfen/IL x Stago and respective Bland-Altman plots using the (**B**) linear and (**C**) non-linear/polynomial model.

*Abbreviations: VWF:Ag, von Willebrand factor antigen*

**SUPPLEMENTARY TABLES**

**Supplementary Table 1**

| ***Patient characteristics*** | **Overall, n=353** |
| --- | --- |
| **Age, years, mean (SD)** | 56.2 (11.9) |
| **Sex, n (%)** | |
| Male | 243 (68.8%) |
| Female | 110 (31.2%) |
| **Aetiology of liver disease, n (%)** | |
| Alcohol-related liver disease | 175 (49.6%) |
| Viral | 61 (17.3%) |
| Alcohol-related liver disease/Viral | 25 (7.1%) |
| Metabolic-dysfunction associated steatotic liver disease | 19 (5.4%) |
| Cholestatic | 18 (5.1%) |
| Other aetiologies | 55 (15.6%) |
| **Removal of the primary aetiological factor at study inclusion, n (%)** | 164 (46.5%) |
| **Viraemic at study inclusion, n (%)** | 33 (9.3%) |
| **Removal of the primary aetiological factor during follow-up, n (%)** | 33 (9.3%) |
| **Type 2 diabetes mellitus, n (%)** | 47 (13.3%) |
| **Anticoagulation at study inclusion, n (%)** | 14 (4.0%) |
| **Antiplatelet therapy at study inclusion, n (%)** | 24 (6.8%) |
| **Hepatocellular carcinoma within Milan criteria, n (%)** | 32 (9.1%) |
| **Characterisation of portal hypertension** | |
| **History of decompensation, n (%)** | 232 (65.7%) |
| **History of variceal bleeding, n (%)** | 47 (13.3%) |
| **Ascites, n (%)** | 206 (58.4%) |
| **Hepatic encephalopathy, n (%)** | 79 (22.4%) |
| **Hepatic venous pressure gradient, mmHg, median (IQR)** | 17.0 (12.0-21.0) |
| **Clinically significant portal hypertension, n (%)** | 310 (87.8%) |
| **Liver stiffness measurement, kPa, median (IQR)** | 36.3 (19.9-62.2) |
| **Continuous attenuation parameter, dB/m, median (IQR)** | 225 (178-270) |
| **EASL stages, n (%)** | |
| 0 | 29 (8.2%) |
| 1 | 43 (12.2%) |
| 2 | 49 (13.9%) |
| 3 | 18 (5.1%) |
| 4 | 123 (34.8%) |
| 5 | 91 (25.8%) |
| **Varices, n (%)** | |
| None | 136 (38.5%) |
| Small | 85 (24.1%) |
| Large | 129 (36.5%) |
| **Red spot sign, n (%)** | 42 (11.9%) |
| **Portal hypertensive gastropathy, n (%)** | |
| None | 93 (26.3%) |
| Mild | 170 (48.2%) |
| Severe | 47 (13.3%) |
| **Liver disease scores** | |
| **Child-Turcotte-Pugh score, points, median (IQR) and n (%)** | 7 (5-9) |
| A | 149 (42.2%) |
| B | 147 (41.6%) |
| C | 57 (16.1%) |
| **MELD score, points, median (IQR)** | 12 (9-16) |
| **MELD-Na score, points, median (IQR)** | 14 (10-18) |
| **UNOS MELD (2016) score, points, median (IQR)** | 12 (9-16) |
| **Laboratory parameters** | |
| **Haemoglobin, g/dL, median (IQR)** | 11.4 (9.8-12.9) |
| **Platelet count, G/L, median (IQR)** | 100 (69-144) |
| **White blood cell count, G/L, median (IQR)** | 4.62 (3.22-6.19) |
| **Sodium, mmol/L, mean (SD)** | 137.0 (3.9) |
| **Creatinine, mg/dL, median (IQR)** | 0.74 (0.61-0.94) |
| **Bilirubin, mg/dL, median (IQR)** | 1.3 (0.8-2.4) |
| **Albumin, g/L, median (IQR)** | 35.0 (5.9) |
| **Alkaline phosphatase, IU/L, median (IQR)** | 98 (72-128) |
| **Aspartate aminotransferase, IU/L, median (IQR)** | 43 (30-63) |
| **Alanine aminotransferase, IU/L, median (IQR)** | 30 (21-45) |
| **Gamma glutamyltransferase, IU/L, median (IQR)** | 82 (49-176) |
| **INR, %, mean (SD)** | 1.45 (0.33) |
| **Bile acids, µmol/L, median (IQR)** | 24.5 (8.9-49.1) |
| **Ammonia, µmol/L, median (IQR)** | 38.8 (29.9-50.5) |
| **C-reactive protein, mg/dL, median (IQR)** | 0.37 (0.14-0.86) |
| **Interleukin 6, pg/mL, median (IQR)** | 10.4 (5.6-22.9) |
| **Procalcitonin, µg/L, median (IQR)** | 0.12 (0.07-0.19) |
| **Lipopolysaccharid binding protein, mg/L, median (IQR)** | 6.85 (5.08-8.82) |
| **proBNP, pg/mL, median (IQR)** | 113 (50-253) |
| **Copeptin, pmol/L, median (IQR)** | 9.4 (4.4-18.4) |
| **Renin, µIU/mL, median (IQR)** | 35 (11-119) |
| **Enhanced liver fibrosis test, median (IQR)** | 11.7 (10.8-12.8) |

**Supplementary Table 1.** Detailed patient characteristics.

*Abbreviations: ALD, alcohol-related liver disease; EASL, European Association for the Study of the Liver; IQR, interquartile range; MASLD, metabolic dysfunction-associated steatotic liver disease; MELD score, model for end-stage liver disease; n, number; SD, standard deviation; UNOS, United Network for Organ Sharing; VWF:Ag, von Willebrand factor antigen*

**Supplementary Table 2**

| **VWF:Ag levels & VITRO scores** | **Overall, n=353** | **cACLD, n =121** | **dACLD, n=232** | **p-value** |
| --- | --- | --- | --- | --- |
| **VWF:Ag, Stago, %, median (IQR)** | 282 (195-358) | 214 (163-292) | 314 (227-409) | **<0.001** |
| **VWF:Ag, Siemens, %, median (IQR)** | 215 (148-298) | 168 (136-227) | 259 (176-335) | **<0.001** |
| **VWF:Ag, Werfen/IL, %, median (IQR)** | 256 (193-325) | 215 (160-267) | 281 (211-370) | **<0.001** |
| **VITRO w/Stago, median (IQR)** | 2.8 (1.6-4.5) | 2.4 (1.3-3.8) | 2.9 (1.8-5.1) | **<0.001** |
| **VITRO w/Siemens, median (IQR)** | 2.2 (1.3-3.7) | 1.8 (1.1-3.0) | 2.5 (1.4-4.2) | **<0.001** |
| **VITRO w/Werfen/IL, median (IQR)** | 2.5 (1.5-4.2) | 2.2 (1.2-3.2) | 2.8 (1.7-4.8) | **<0.001** |

**Supplementary Table 2.** Distribution of VWF:Ag/VITRO values according to disease stage at study inclusion.

*Abbreviations: IQR, interquartile range; VITRO, ratio of von Willebrand factor antigen and platelets; VWF:Ag, von Willebrand factor antigen*

**Supplementary Table 3**

| **Metric** | **Siemens** | **Werfen/IL** |
| --- | --- | --- |
| **ICC** | 0.921 | 0.911 |
| **Pearson’s r** | 0.922 | 0.914 |
| **R^2^** | 0.850 | 0.835 |
| **Intercept (SE)** | 58.770 (6.041) | 55.136 (6.444) |
| **Slope (SE)** | 0.963 (0.022)* | 0.848 (0.020)* |
| **Coefficient of variation** | 0.916 | 1.179 |
| **Repeatability coefficient** | 143.946 | 121.727 |
| **Repeatability coefficient in %** | 56.8% | 40.5% |
| **Within-subject coefficient of variation** | 0.042 | 0.021 |
| **Mean difference (95% CI) ≙ mean calibration** | 49.6 (43.9, 55.3) | 12.1 (5.7, 18.5) |
| **Interval of agreement** | 212.7 | 239.1 |
| **Lower limit of agreement (95% CI)** | -56.8 (-66.5, -47.1) | -107.5 (-118.4, -96.6) |
| **Upper limit of agreement (95% CI)** | 155.9 (146.2, 165.6) | 131.7 (120.7, 142.6) |
| **Residuals, median** | -9.65 | 0.17 |
| **Jarque-Bera normality test, X^2^** | 810.034* | 966.616* |
| **Durbin-Watson test** | 1.581* | 1.900 |

* denotes p<0.05

Intraclass correlation coefficient quantifies the degree of agreement between measurements. Pearson’s r measures the strength and direction of a linear relationship between continuous variables. R^2^ coefficient of determination, Pearson’s r squared. Intercept means the predicted value of the dependent variable on the linear regression coefficient the slope represents the estimated average change in the dependent variable. Coefficient of variation represents the relative variability that quantifies the dispersion of the data relative to its mean, calculated as the ratio of the standard deviation to the mean. The repeatability coefficient provides the agreement between two measurements of the same quantity taken under identical conditions. The within-subject coefficion of variation measures the reproducibility of repeated measurements on the same individual, calculated as the ratio of the within-subject standard deviation to the mean of measurements. The mean difference, interval of agreement, lower and upper limit of agreement, and resduals are measures derived from the Bland-Altman plot, assessing the agreement between two different measurement methods by measuring the difference between the two measurements against their average. The Jarque-Bera normality test is a ‘goodness-of-fit’ test that checks if a dataset's skewness and kurtosis match those of a normal distribution. The Durbin-Watson test checks for an auto-correlation, a correlation between the residuals.

**Supplementary Table 3.** Inter-assay comparison of VWF:Ag using different test for agreement.

*Abbreviations: CI, conficence interval; ICC, intraclass correlation coefficient; SE, standard error; VWF:Ag, von Willebrand factor antigen*

**Supplementary Table 4**

| **Further hepatic decompensation/liver-related death** | **Stago (ref)** | | **Siemens** | | **Werfen/IL** |
| --- | --- | --- | --- | --- | --- |
| **HR (95%CI), per 10** | 1.02 (1.00-1.03), p=0.012 | | 1.02 (1.00-1.03), p=0.017 | | 1.01 (1.00-1.03), p=0.012 |
| **Concordance (SE)** | 0.561 (0.031) | | 0.554 (0.030)* | | 0.568 (0.030)* |
| **Time-dependent AUROC** | | **12 months** | | **24 months** | |
| **Events** | | 75 | | 94 | |
| **Stago (ref)** | | 0.553 | | 0.594 | |
| **Siemens** | | 0.539^#^ | | 0.592^#^ | |
| **Werfen/IL** | | 0.557^#^ | | 0.605^#^ | |
| **Further hepatic decompensation/liver-related death** | **VITRO-Stago (ref)** | | **VITRO-Siemens** | | **VITRO-Werfen/IL** |
| **HR (95%CI), per 10** | 1.04 (0.99-1.10), p=0.115 | | 1.05 (0.99-1.11), p=0.068 | | 1.05 (0.99-1.10), p=0.079 |
| **Concordance (SE)** | 0.541 (0.030) | | 0.535 (0.031) | | 0.542 (0.030) |
| **Time-dependent AUROC** | | **12 months** | | **24 months** | |
| **Events (ref)** | | 75 | | 94 | |
| **VITRO-Stago** | | 0.557 | | 0.561 | |
| **VITRO-Siemens** | | 0.543^#^ | | 0.560^#^ | |
| **VITRO-Werfen/IL** | | 0.556^#^ | | 0.567^#^ | |

^#^ p-value >0.05

**Supplementary Table 4.** Predictive ability of VWF:Ag/VITRO levels for further hepatic decompensation in patients with decompensated cirrhosis (n=232) according to the applied assay.

*Abbreviations: AUROC, area under the receiver operating curve; cACLD, compensated advanced chronic liver disease; CI, confidence interval; SE, standard error; VITRO, ratio of von Willebrand factor antigen and platelet count; VWF:Ag, von Willebrand factor antigen*
